# Neurological Presentations in Patients with COVID-19 in Cytokine Storm

**DOI:** 10.1017/cjn.2021.247

**Published:** 2021-10-29

**Authors:** Gorkem Tutal Gursoy, Hatice Yuksel, Inci Mulkem Simsek, Saniye Oral, Fadime Erdogan Kucukdagli, Ayberk Karaman, Esragul Akinci, Aliye Bastug, Hatice Rahmet Guner, Hesna Bektas

**Affiliations:** 1 Department of Neurology, Ankara City Hospital, Cankaya, Turkey; 2 Department of Infectious Diseases and Clinical Microbiology, Ankara City Hospital, Cankaya, Turkey; 3 Department of Neurosurgery, Ankara City Hospital, Cankaya, Turkey

**Keywords:** COVID-19, Cytokine storm, Neurological disorders, Altered level of consciousness, Interleukin-6

## Abstract

**Background:**

Coronavirus disease 2019 (COVID-19) infection causes a wide variety of neurological disorders by affecting both central and peripheral nervous systems. The cytokine storm (CS) has been blamed for the development of severe neurological disorders in COVID-19. However, the relationship between COVID-19 CS and neurological manifestations has not been adequately studied. Thus, we aimed to investigate the neurological presentations in patients with COVID-19 CS.

**Methods:**

The study population consisted of hospitalized moderate-to-severe COVID-19 patients. It was divided into two groups CS (36 patients, 29.3%) and non-CS (87 patients, 70.7%) based on significant clinical symptoms, elevated inflammatory marker levels, radiological findings, and interleukin-6 levels (IL-6).

**Results:**

The three most common neurological symptoms in the CS group were altered level of consciousness, headache, and unsteadiness. Altered level of consciousness was higher in the CS group (69.4%) than the non-CS group (25.3%) (*p*:0.001). The frequency of headache was comparable in both groups (*p*:0.186). The number of patients requiring intensive care unit and intubation was higher in the CS group (*p*:0.005 and *p*:0.001). The mortality rate in the CS group (38.9%) was higher than the non-CS group (8.0%) (*p*:0.001). IL-6, CRP, ferritin, neutrophil-lymphocyte ratio, procalcitonin, and D-dimer levels were higher in the CS group (for all *p*:0.001) while lymphocyte count was lower (*p*:0.003).

**Conclusion:**

The most common neurological presentation in patients with CS was altered level of consciousness. The presence of CS was an independent risk factor for high mortality.

## RÉSUMÉ

Symptômes neurologiques chez des patients atteints d’une infection à la COVID-19 et victimes d’une tempête de cytokines

**Contexte:**

Les infections à la COVID-19 peuvent provoquer une grande variété de troubles neurologiques en affectant le système nerveux central et le système périphérique. Les tempêtes de cytokines (TC) ont ainsi été mises en cause dans le développement de graves troubles neurologiques liés aux infections à la COVID-19. Cela dit, l’association entre ces infections (et les TC) et des symptômes d’atteinte neurologique n’a pas encore été étudiée de façon adéquate. Voilà pourquoi nous entendons nous pencher sur de tels symptômes chez des patients atteints d’une infection à la COVID-19 qui ont été victimes d’une TC.

**Méthodes:**

La cohorte étudiée était composée de patients atteints d’une infection à la COVID-19 modérée à sévère ayant été hospitalisés. Elle a été divisée en deux sous-cohortes sur la base de symptômes cliniques significatifs, de taux élevés de marqueurs inflammatoires, de résultats radiologiques et de taux d’interleukine-6 (IL-6) : d’une part, une sous-cohorte de 36 patients victimes d’une TC (29,3 %) et, d’autre part, une autre regroupant 87 autres patients ayant été épargnés par ce phénomène (70,7 %).

**Résultats:**

Les trois symptômes neurologiques les plus fréquents dans la première sous-cohorte étaient une altération des niveaux de conscience, des maux de tête et un déséquilibre. Des niveaux de conscience altérés se sont ainsi révélés plus élevés au sein de la sous-cohorte des patients victimes d’une TC (69,4 %) qu’au sein de l’autre sous-cohorte (25,3 %) (*p* = 0,001). La fréquence des maux de tête s’est quant à elle avérée comparable d’une sous-cohorte à l’autre (*p* = 0,186). Soulignons que le nombre de patients nécessitant des soins intensifs et l’intubation était plus élevé dans la première sous-cohorte (*p* = 0,005 et *p* = 0,001). Le taux de mortalité dans cette dernière (38,9 %) était également plus élevé que dans la deuxième sous-cohorte (8,0 %) (*p* = 0,001). Enfin, les taux d’IL-6, de protéine C réactive (PCR), de ferritine, de procalcitonine (PCT) et de D-dimères, de même que le rapport neutrophiles-lymphocytes, étaient plus élevés dans la première sous-cohorte (tous les *p* = 0,001) tandis que la numération lymphocytaire était plus faible (*p* = 0,003).

**Conclusion:**

Le symptôme neurologique le plus courant chez les patients victimes d’une TC était une altération du niveau de conscience. Le fait d’être victime d’un tel choc inflammatoire a aussi été identifié comme un facteur de risque indépendant de mortalité élevée.

## Introduction

Coronavirus disease 2019 (COVID-19) is an infectious disease caused by severe acute respiratory syndrome coronavirus 2 (SARS-CoV-2), responsible for the deaths of millions of people since it was declared a pandemic by the World Health Organization.^1^ Although COVID-19 primarily involves the respiratory system, it often tends to spread in various organ systems.^2^

As a part of many organ system involvements, COVID-19 also causes serious disorders related to both the central and peripheral nervous systems.^2^ However, the pathogenesis of neurological involvement has not been clearly demonstrated. Recently, neurological system involvement has been partially attributed to cytokine storm (CS).^3,4^

CS is an inclusive term describing various disorders with hyperinflammation and multiorgan involvement characterized by excessive cytokine release resulting from an exaggerated immune activation.^5,6^ CS can occur during the course of autoimmune diseases, malignancies, and various infectious diseases such as SARS, MERS, and H5N1 influenza.^4,6^

Excessive increases in levels of cytokines such as interleukin-1β, interleukin-6 (IL-6), IP-10, IL-2R, tumor necrosis factor-α, and interferon-γ have been shown in CS. However, their role in the pathogenesis of CS has not been fully elucidated.^6,7^ Recently, IL-6, a cytokine secreted by immune and stromal cells, has become the focus of interest in COVID-19 CS. It is a proinflammatory cytokine and plays a key role in CS by amplifying the immune response.^8^ The level of IL-6 elevation correlates with the need for mechanical ventilation and increased mortality.^9^

**Figure 1:**
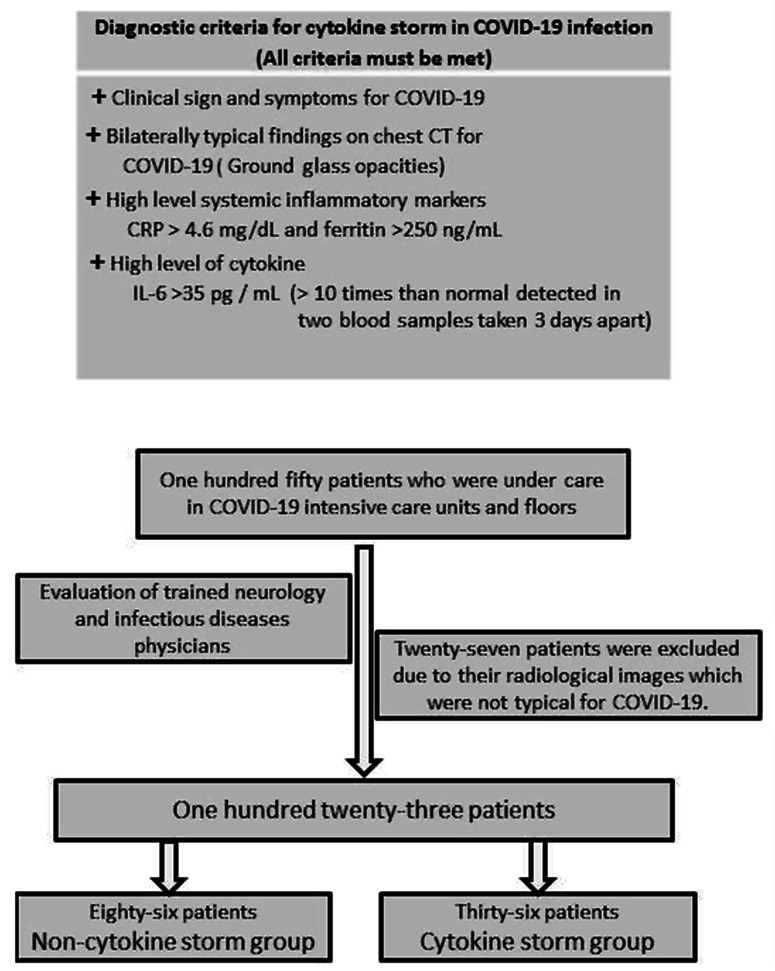
Modified temple criteria for COVID-19 cytokine storm (with permission of Professor Roberto Carrichio) and patient selection.

Accumulating data suggest that a subset of severe COVID-19 patients has CS. However, the exact criteria for the diagnosis of COVID-19 CS have not yet been established.^10,11^ Caricchio et al.^10^ described some predictive criteria for the diagnosis of COVID-19-associated CS in their well-designed study. It was called the Temple Criteria.^12^ However, they did not use any cytokine level as a criterion (Figure 1).

In this study, we aimed to investigate the neurological presentations in patients with COVID-19 CS by using modified Temple criteria.

## Patients and Methods

This prospective study was conducted in Ankara City Hospital. One-hundred fifty patients under care in COVID-19 intensive care units and floors were enrolled in the study.

We recorded patients’ information, including age, gender, medical comorbidities, and neurological comorbidities. Laboratory tests including IL-6, CRP, ferritin, procalcitonin, D-dimer, fibrinogen, troponin I, complete blood count, creatinine, liver function tests, and creatine kinase were studied. A polymerase chain reaction (RT-PCR) test for COVID-19 from nasopharyngeal swab was studied. All patients had chest computed tomography.

Patients’ neurological symptoms including headache, altered level of consciousness, seizures, vertigo, unsteadiness, anosmia/hyposmia, dysgeusia, nausea, paresthesia, tinnitus, and sleep disorders were obtained by trained neurologists from the patients or their relatives and medical notes (recorded during hospital follow-up prospectively). Altered level of consciousness was defined as changes in consciousness such as somnolence, agitation, delirium, confusion, and coma. The evaluation of unsteadiness, which is defined as difficulty in standing and inability to maintain body balance, was performed when the patients’ consciousness level was normal.

The patients’ clinical, laboratory, and radiological data were evaluated by a team consisting of trained neurology and infectious diseases physicians. “Confirmed cases” with SARS-CoV-2 infection detected by RT-PCR test of a nasopharyngeal sample or confirmed antibody test were included. In addition, “probable cases” of SARS-CoV-2 infection with severe respiratory disease with clinical and radiographic evidence of pneumonia despite a negative RT-PCR or antibody testing were included in the study.^13^ Twenty-seven patients with negative RT-PCR tests were excluded from the study because their radiological images were not typical for COVID-19.

COVID-19 CS is defined as a state of hyperinflammation characterized by excessive cytokine release in a subset of patients with COVID-19.^14^ To date, no valid criteria have been defined for the COVID-19 CS. For the first time, Caricchio et al. established criteria to define COVID-19 CS. Their criteria were based on the presence of COVID-19 symptoms, ground-glass opacities (GGO) detected on lung images, elevated CRP, and ferritin levels accompanied by some abnormal biochemical and hematological parameters.^10^ In the identification of patients with CS, we used some criteria defined by Caricchio et al. and also IL-6 levels as an additional criterion.^10^ IL-6 is recognized as a key cytokine driving the hyperinflammatory state in COVID-19 CS.^8,15^ Therefore, we thought it would be beneficial to add IL-6 level as a criterion to the COVID-19 CS criteria. There has no generally accepted cut-off value of IL-6 for the diagnosis of CS in COVID-19 infection. However, a tenfold high level of IL-6 was found to be associated with the severity of the disease.^16,17^ Therefore, we considered IL-6 levels 10 times higher than normal detected in two blood samples taken 3 days apart from the patients as an indicator of ongoing excessive cytokine release and used it as an additional criterion for CS.

Thirty-six patients (29.3%) were included in the CS group based on the criteria of prominent clinical symptoms (cough, fever, respiratory distress, and so forth), high inflammatory marker levels (CRP > 4.6 mg/dL and ferritin > 250 ng/mL), typical radiological findings (bilateral GGO in the lungs), and high cytokine level (IL-6 levels > 10 times than normal detected in two blood samples taken 3 days apart). The remaining 87 patients (70.7%) were enrolled in the non-CS group.

The study was approved by the local ethical committee *(*Ankara City Hospital Ethics Committee).

All statistical analyses were done using IBM SPSS statistic 22.0 (Chicago, IL, USA). Data were expressed as mean ± SD. Continuous variables were compared using the Mann−Whitney *U*-test. Categorical variables were compared using the chi-square test. Binary logistic regression analysis was performed to detect independent factors associated with altered level of consciousness and mortality. A *p*-value < 0.05 was considered statistically significant.

## Results

The mean age in the CS group was higher than in the non-CS group (*p*:0.003). The CS group included more males than the non-CS group (*p*:0.027). RT-PCR positive for COVID-19 was similar in both groups (*p*:0.254) (Table 1).

**Table 1:** Comparison of the demographic data of patients with and without cytokine storm

	Non-cytokine storm group n:87	Cytokine storm group n:36	*p*
Age (years)	59.67 ± 13.66	67.52 ± 10.89	**0.003**
Gender Female/Male	43 (49.4%)/44 (50.6%)	10(27.8%)/26 (72.2%)	**0.027**
RT-PCR for COVID-19			
Positive	67 (68.4%)	31 (86.1%)	0.254
Negative	20 (23.0%)	5 (13.9%)	
Medical comorbidity	56 (64.4%)	26 (72.2%)	0.400
Coronary artery disease	14 (16.1%)	12 (33.3%)	0.033
Arrhythmia	5 (5.2%)	–	0.142
Hypertension	44 (50.6%)	21 (58.3%)	0.433
Hyperlipidemia	12 (13.8%)	5 (13.9%)	0.989
Diabetes mellitus	25 (28.7%)	12 (33.3%)	0.613
Chronic pulmonary disease	8 (9.2%)	7 (19.4%)	0.114
Chronic renal failure	2 (2.3%)	3 (8.3%)	0.123
Neurological comorbidity	15 (17.2%)	10 (27.8%)	0.186
Cerebrovascular accident	11 (12.6%)	4 (11.1%)	0.813
Dementia	4 (4.6%)	4 (11.1%)	0.231
Epilepsy	4 (4.2%)	–	0.575
Parkinson disease	1 (1.1%)	1 (2.8%)	0.501

If a *p* value is < 0.05, it was written in bold to emphasize.

The neurological and medical comorbidities in the CS and non-CS groups are presented in Table 1. Although the presence of neurological comorbidity was more frequent in the CS group than in the non-CS group, the difference was not significant (*p*:0.186). The presence of medical comorbidity was similar in both groups (*p*:0.400) (Table 1).

IL-6, CRP, ferritin, NLR, neutrophil count, procalcitonin, D-dimer, troponin I, creatinine, AST, LDH, and CK level were higher in the CS group than non-CS group (*p*:0.001, *p*:0.001, *p*:0.001, *p*:0.00 *p*:0.046, *p*:0.001, *p*:0.001, *p*:0.018, *p*:0.008, *p*:0.010, *p*:0.001, and *p*:0.003, respectively) while lymphocyte count was lower (*p*:0.003) (Table 2).

**Table 2:** Comparison of the laboratory data of patients with and without cytokine storm

	Non-cytokine storm group n:87	Cytokine storm group n:36	*p*
Interleukine-6 (pg/mL) (1. measurement)	30.70 ± 35.48	122.85 ± 163.44	**0.001**
Interleukine-6 (pg/mL) (2. measurement)	117.44 ± 248.69	570.80 ± 432.78	**0.001**
C-reactive protein (mg/dL)	10.68 ± +9.09	17.31 ± 10.37	**0.001**
Ferritin (ng/mL)	572.63 ± 554.98	2882.86 ± 7069.34	**0.001**
Neutrophil-lymphocyte ratio	8.49 ± 20.79	10.78 ± 10.76	**0.001**
Neutrophil (10^9^/L)	6.79 ± 11.94	7.53 ± 7.03	**0.046**
Lymphocyte (10^9^/L)	1.56 ± 2.32	0.87 ± 0.44	**0.003**
Hemoglobin (g/dL)	13.08 ± 1.49	12.56 ± 1.99	0.503
Platelets (10^9^/L)	259.56 ± 121.47	247.02 ± 108.45	0.691
Procalcitonin (µg/L)	0.17 ± 0.39	4.19 ± 14.17	**0.001**
D-dimer (mg/dL)	1.47 ± 2.64	8.21 ± 13.44	**0.001**
Fibrinogen (g/L)	5.28 ± 1.77	5.62 ± 1.94	0.231
Troponin I (ng/L)	12.31 ± 26.95	215.75 ± 711.97	**0.018**
Creatinine (mg/dL)	0.81 ± 0.27	1.34 ± 1.31	**0.008**
Alanine aminotransferase (U/L)	48.49 ± 60.07	163.61 ± 524.46	0.123
Aspartate aminotransferase (U/L)	52.80 ± 75.44	439.22 ± 1596.71	**0.010**
Lactate dehydrogenase (U/L)	343.13 ± 125.82	1006.80 ± 2164.36	**0.001**
Creatine kinase (U/L)	215.77 ± 469.82	617.08 ± 912.03	**0.003**

If a *p* value is < 0.05, it was written in bold to emphasize.

The number of patients requiring intensive care unit and intubation was higher in the CS group than in the non-CS group (*p*:0.005 and *p*:0.001). Mortality rate in the CS group (38.9%) was higher than in the non-CS group (8.0%) (*p*:0.001) (Table 3).

**Table 3:** Comparison of the clinic data of patients with and without cytokine storm

	Non-cytokine storm group n:87	Cytokine storm group n:36	*p*
Intensive care unit	36 (41.4%)	25 (69.4%)	**0.005**
Intubation	11 (12.6%)	14 (38.9%)	**0.001**
Mortality	7 (8.0%)	14 (38.9%)	**0.001**
Neurological presentations			
Altered level of consciousness	22 (25.3%)	25 (69.4%)	**0.001**
Headache	57 (65.5%)	19 (52.8%)	0.186
Seizure	4 (4.6%)	3 (8.3%)	0.416
Dizziness	29 (33.3%)	9 (25.0%)	0.363
Vertigo	26 (29.9%)	6 (16.7%)	0.128
Unsteadiness	16 (18.4%)	11 (30.6%)	0.138
Anosmia/Hyposmia	9 (10.3%)	3 (8.3%)	0.732
Dysgeusia	19 (10.3%)	6 (16.7%)	0.369
Nausea	25 (28.7%)	9 (25.0%)	0.673
Paresthesia	12 (13.8%)	54 (13.9%)	0.989
Tinnitus	3 (3.4%)	–	0.555
Sleep disorders	11 (12.6%)	4 (11.1%)	0.813
Ischemic stroke	11 (12.6%)	4 (11.1%)	0.813
Hemorrhagic stroke	2 (2.3%)	–	0.359
Cerebral vein thrombosis	1 (1.1%)	–	0.518

If a *p* value is < 0.05, it was written in bold to emphasize.

The three most common neurological symptoms in CS group were altered level of consciousness, headache, and unsteadiness. While the frequency of headache was the same in both groups (*p*:0.186), altered level of consciousness was higher in the CS group (69.4%) than the non-CS group (25.3%) (*p*:0.001). Although the frequency of unsteadiness in the CS group (30.6%) was higher than in the non-CS group (18.4%), there were no significant differences (*p*:0.138). Ischemic stroke was observed more frequently than hemorrhagic stroke in both groups; however, there was no difference between the two groups regarding the frequency of ischemic stroke (*p*:0.638). There was no difference between the two groups in terms of the frequency of all other neurological disorders (Table 3).

In binary logistic regression analysis, CS and neurological comorbidity were determined as an independent risk factor for altered level of consciousness. CS and age were detected as independent risk factors for mortality (Table 4).

**Table 4:** Independent factors associated with altered level of consciousness and mortality in binary logistic regression analysis

	Altered level of consciousness		
	*p* value	Exp(B)	%95 Confidence interval
Age	0.901	1.002	0.966–1.040
Gender	0.316	1.570	0.651–3.787
Cytokine storm	**0.001**	6.059	2.365–15.522
Neurological comorbidity	**0.038**	3.087	1.065–8.951
Medical comorbidity	0.083	2.413	0.891–6.536

	Mortality		
	*p* value	Exp(B)	%95 Confidence interval
Age	**0.019**	1.067	1.011–1.126
Gender	0.622	1.333	0.425–4.175
Cytokine storm	**0.003**	5.260	1.778–15.556
Neurological comorbidity	0.666	0.755	0.211–2.698
Medical comorbidity	0.402	1.739	0.477–6.342

If a *p* value is < 0.05, it was written in bold to emphasize.

## Discussion

We demonstrated that the most common neurological disorder in patients with COVID-19 CS is altered level of consciousness. CS was associated with increased need for intensive care and a high mortality rate. CS and age were independent risk factors for mortality in COVID-19 patients. In COVID-19 patients without CS, the most common neurological symptom was headache. Their mortality rate was also very low compared to those with CS.

COVID-19 affects both the peripheral and central nervous system (CNS) intensely as well as other organ systems. It can cause a wide range of problems in patients, from mild symptoms such as headache and loss of smell to severe symptoms such as impaired consciousness and epileptic seizures.^2,18^ While it causes mild neurological symptoms in most patients, the underlying pathogenesis of why it causes severe neurological disorders in a subgroup of patients is not fully known.^2,19^

The results of recent studies suggest that CS may be responsible for the severe involvement seen in both the neurological and other systems in COVID-19. CS may occur during the course of infections, neoplastic, and autoimmune diseases. This hypercytokinemic inflammation may be responsible for end-organ damage.^7,19,20^ Well-established criteria are available for the determination of two CS forms containing hemophagocytic lymphohistiocytosis (HLH) and macrophage activation syndrome (MAS). Although some findings of CS in COVID-19 are similar to CS in HLH and MAS, it has unique features different from them.^7,10^ Caricchio et al.^10^ showed that the criteria defined for HLH and MAS were not useful in the diagnosis of COVID-19 CS.

The exact criteria for the diagnosis of COVID-19 CS have not yet been clarified. Therefore, a limited number of studies in the literature provide information about the neurological involvement in patients with COVID-19 CS. Although previous studies revealed a high frequency of impaired consciousness in severe COVID-19 infection,^18,21^ its frequency in COVID-19 CS has not been reported before. Our study revealed a very high frequency of altered level of consciousness (69.4%) in patients with CS. Impaired consciousness in COVID-19 occurs as a result of factors such as metabolic-toxic, hypoxic, cerebrovascular events, and medications.^22^ Direct invasion of the CNS by the virus has also been implicated as a cause of neurological symptoms in COVID-19.^23^ Consistent with this mechanism, SARS-CoV-2 was detected in brain specimens in an autopsy series involving 22 patients.^24^ However, in a larger autopsy series involving 43 patients, neuroimmune activation was detected in all brain specimens examined. The authors showed that there was no correlation between the severity of neuroimmune activation and the presence of SARS-CoV-2 in the brain specimens. They stated that the detected neuropathological changes may be due to other factors such as CS and neuroimmune stimulation rather than direct invasion of the virus into the CNS.^25^ In a recent study, increased levels of cytokines in cerebrospinal fluid have been demonstrated in COVID-19 patients with encephalitis.^3^ Thus, one of the reasons for impaired consciousness in COVID-19 may be the direct effect of increased cytokine levels on the CNS.^3,4^

SARS-CoV-2 has been shown to cause endothelitis, which might explain the increased rate of thrombosis in various organs. In addition, cytokines can cause significant endothelial damage.^26,27^ Also, proinflammatory cytokines, including IL-6, activate the coagulation system and inhibit the anticoagulant pathway. Therefore, there is a hypercoagulable condition in COVID-19 CS.^4,7^ We found a high frequency of ischemic stroke in our study population, consistent with the literature. Hypothetically, we expected a higher frequency of ischemic stroke in patients with CS, but we found a similar frequency of ischemic stroke in patients with and without CS. Pathology studies have shown an increased incidence of microthrombi in patients with severe COVID-19.^28,29^ However, microthrombi may not be demonstrated on radiological images. For this reason, our study design was insufficient to demonstrate and compare the exact frequency of microangiopathic thrombus in patients with and without CS. Consequently, we cannot exclude the possibility that one of the underlying causes of the impaired consciousness’ high frequency in our CS patients may be cytokine-induced microthrombi.

Hemorrhagic stroke is a rare complication of COVID-19.^30^ The results of our study were consistent with the literature. While hemorrhagic stroke was not detected in any patient with CS, it was detected in only two patients without CS.

One of the most common complaints in COVID-19 patients is a headache. The frequency of headache in hospitalized patients with COVID-19 has been reported to be 13%–34%. Stimulation of peripheral trigeminal nerve endings by factors such as direct invasion of the virus, hypoxia, and pro-inflammatory cytokines is blamed for the occurrence of headache, but the exact mechanism is still unknown.^4,31^ Although the occurrence of headache in COVID-19 is attributed to CS, a direct connection has not been established.^4^ We also did not find a causal relationship between headache and CS. In our study, while headache was the second most common symptom in patients with CS, it was the most common symptom in patients without CS. Since we asked our patients about their headaches as a part of standard questioning, reporting mild headaches by patients may explain the high frequency of headaches in our study group.

Epileptic seizure frequency has been reported between 1.6% and 4% in COVID-19.^21,32^ Although there is no defined mechanism, CS has also been associated with the occurrence of seizures. Increased IL-6 levels have been associated with the development of seizures.^33^ We found a higher frequency of seizures in patients with CS. Due to the small number of our study population, that result may not be generalized.

Our study suggests that CS does not have a significant effect on some neurological presentations such as dysgeusia, anosmia, nausea, and dizziness. However, considering the limited data in our study, large and detailed studies are needed to clearly define the relationship between them.

As discussed above, IL-6 elevation also has been demonstrated in severe neurological involvement as in other organ systems affected by hypercytokinemic inflammation.^4,7,14^ This excess IL-6 release can play a major role in the pathogenesis or simply be an epiphenomenon of other cytokine releases.^34^ As a result, IL-6 stands out as an important cornerstone indicating serious organ involvement in CS. Therefore, the addition of cytokines, especially IL-6, as a criterion in the diagnosis of CS, may help in establishing an accurate diagnosis.

To our knowledge, our study is the first to present neurological findings specifically in COVID-19 CS, although it is difficult to follow the rapidly growing number of COVID-19 related studies. The limitation of our study is that only one cytokine (IL-6) was studied to define COVID-19 CS. Another limitation of our study is that assessment of consciousness level may have subjectivity in the acute care settings.

In conclusion, we detected CS in approximately one-third of hospitalized moderate-severe COVID-19 patients. Altered level of consciousness was the most common neurological complication in patients with CS. Also, the presence of CS was an independent risk factor for its high mortality rate.
